# Neural Differentiation of Mouse Embryonic Stem Cells—An *in vitro* Approach to Profile DNA Methylation of Reprogramming Factor Sox2-SRR2

**DOI:** 10.3389/fgene.2021.641095

**Published:** 2021-03-22

**Authors:** Sajida Batool, Mahmood Akhtar Kayani, Martin Valis, Kamil Kuca

**Affiliations:** ^1^Cancer Genetics and Epigenetics Laboratory, Department of Biosciences, COMSATS University Islamabad, Islamabad, Pakistan; ^2^Department of Neurology of the Medical Faculty of Charles University and University Hospital in Hradec Kralove, Hradec Kralove, Czechia; ^3^Department of Chemistry, University of Hradec Kralove, Hradec Kralove, Czechia

**Keywords:** embryonic stem cells, SOX2, SRR2, neural differentiation, DNA methylation, epigenetic regulation, pluripotency, mouse

## Abstract

Sox2 is one of the core transcription factors maintaining the embryonic stem cells (ES) pluripotency and, also indispensable for cellular reprogramming. However, limited data is available about the DNA methylation of pluripotency genes during lineage-specific differentiations. This study investigated the DNA methylation of Sox2 regulatory region 2 (SRR2) during directed differentiation of mouse ES into neural lineage. ES cells were first grown to form embryoid bodies in suspension which were then dissociated, and cultured in defined medium to promote neural differentiation. Typical neuronal morphology together with the up-regulation of Pax6, neuroepithelial stem cell intermediate filament and β-tubulin III and, down-regulation of pluripotency genes Oct4, Nanog and Sox2 showed the existence of neural phenotype in cells undergoing differentiation. Three CpGs in the core enhancer region of neural-specific SRR2 were individually investigated by direct DNA sequencing post-bisulfite treatment and, found to be unmethylated in differentiated cells at time-points chosen for analysis. This analysis does not limit the possibility of methylation at other CpG sites than those profiled here and/or transient methylation. Hence, similar analyses exploring the DNA methylation at other regions of the Sox2 gene could unravel the onset and transitions of epigenetic signatures influencing the outcome of differentiation pathways and neural development. The data presented here shows that *in vitro* neural differentiation of embryonic stem cells can be employed to study and characterize molecular regulatory mechanisms governing neurogenesis by applying diverse pharmacological and toxicological agents.

## Introduction

Sox proteins are instrumental for embryonic development chiefly because of their multifaceted regulatory functions in cellular differentiation pathways. Their influence on cell specification is mainly through their participation as transcriptional factors and support component of chromatin structure (Pevny and Lovell-Badge, 1997). They were initially recognized because of their DNA-binding domain (DBD) being highly homologous with sex determining factor known as Sry-HMG box present on Y-chromosome in both mouse and humans (Gubbay et al., 1990; Sinclair et al., 1990). The proteins in Sox family are grouped based on a minimum of 50% sequence similarity of HMG-box with mouse Sry-HMG. This family has 20 different proteins as of present and, they are further categorized into subfamilies (Bowles et al., 2000; Schepers et al., 2002).

Sox2 is a transcription factor of the Sox family of proteins. Expression of Sox2 in mouse has been reported to be regulated both temporally and spatially, i.e., expression is seen all over the inner mass cell (ICM) at first and then is confined to primitive ectoderm, the lens, primordial gut and developing central nervous system (Collignon et al., 1996; Wiebe et al., 2000; Que et al., 2007). Murine Sox2 is an intron-less gene of 2.4 Kb located at 3 A2-B locus made up of 319 amino acids protein, sharing similarity of about 98% with human SOX2 (Collignon et al., 1996). Sox2 is a protein which is regarded as activating member of Sox-gene family (Uchikawa et al., 1999).

Sox2 expression is found to be regulated by different gene-specific enhancers during embryogenesis and, those are significantly conserved among vertebrates (Uchikawa et al., 2003; Kamachi et al., 2009). Sox2 regulatory region 2 (SRR2) is one such evolutionary conserved gene-distal enhancer located about 4 Kb from transcription start site. Oct4-Sox2 protein complex binds with SRR2 enhancer, a binding essentially required for Sox2 expression in neural stem cells (NSC) and ES (Tomioka et al., 2002; Sikorska et al., 2008). Three more ES specific distal enhancers SRR18, SRR107 and SRR111 have also been identified (Zhou et al., 2014). Region-specific temporal and spatial expression of Sox2 during both embryogenesis and neurogenesis in cells of varying origins is thus directed by these different regulatory regions. For example SRR2 acts as NSC specific enhancer in the region of telencephalon but not in stem cells of spinal cord (Zappone et al., 2000; Miyagi et al., 2006).

Neurogenesis is a process whereby new functional cells are continually formed from neural progenitors and, get incorporated into neuronal networks. This differentiation, maturation and localization of new cells into networks in the brain is a complex process (Ming and Song, 2005, 2011). Studies conducted using animal embryos have formed the basis of the current state of knowledge about vertebrate embryogenesis. Still, the heterogeneity of neuronal cells formed during neurogenesis and technical challenges in extracting enough quantities of homogenous cells for extensive signaling and molecular networks analyses have inhibited their temporal and regional characterization (Suter and Krause, 2008).

ES are isolated from the ICM of blastocyst and, can maintain the multi-lineage differentiation potential during *in vitro* cultures. ES cells have become a well-established system for genetic and epigenetic studies of mammalian system, drug discovery, disease modelling and tissue engineering (Murry and Keller, 2008; Muguruma and Sasai, 2012; Prajumwongs et al., 2016). Due to their multi-lineage differentiation potential, even their artificial counterparts ‘induced pluripotent stem cells (iPS)’, despite some differences with ES, have become equally useful tools for disease modelling and are already being exploited for transplantation studies (Han et al., 2011; Yamanaka, 2012; Pocock and Piers, 2018). Neurons generated from ES and iPS cells in vitro have been shown to integrate and function in hosts upon grafting (Henriques et al., 2019). Mechanistic characterization of complex molecular regulatory networks controlling neurogenesis is now becoming possible using embryonic stem cell derived differentiation systems. It is expected that such systems would not only help in understanding the normal brain development but also, would pave the way toward development of cell-based therapeutics. Such therapies are crucially needed for disorders of central nervous system since significant sections of population mostly aging people are continued to be affected (Soliman et al., 2017).

Sox2, an endogenous transcription factor, together with two others namely Oct4 and Nanog has been now extensively proven to govern the pluripotency of ES, and ectopic expression of all these in the somatic cells can even reprogram them to undifferentiated state (Takahashi and Yamanaka, 2006; Takahashi et al., 2007; Park et al., 2008). Development and differentiation are two different processes orchestrated by a precise and timely control of lineage-determining and lineage-specific genes expression. It thus becomes of paramount importance to understand not only these myriad of regulatory networks operating in the cells but also, the gene-regulatory mechanisms controlling and altering the expression of these transcription factors during development and differentiation. DNA methylation is one such gene-regulatory epigenetic modification generally resulting in imprinting of genome, transposon silencing, tissue-specific genes repression, and inactivation of X-chromosome occurs at position 5 of the Cytosine ring found in CG dinucleotides in mammals almost invariably (Smith and Meissner, 2013). Besides DNA methylation, histone modifications and regulatory RNAs are other gene-regulatory epigenetic mechanisms directing the differentiation of NSCs and, therefore have started to become focus of intense research (Sanosaka et al., 2009; Yao and Jin, 2014). The current research was aimed to profile the onset of DNA methylation signatures during targeted differentiation of mouse ES. The work described here has mainly investigated the DNA methylation of a regulatory region of Sox2 namely SRR2 in mouse ES to find the role of methylation of this region in maintaining and/or influencing the differentiation potential of embryonic stem cells. SRR2 has been implicated for Sox2 expression in both undifferentiated cells and NSCs and is highly homologous to human equivalent of SOX2 gene. It is therefore plausible that regulatory mechanisms may as well be conserved and thus, making this region a good candidate for such investigations. This is the first study to our knowledge attempting to profile DNA methylation of SRR2 region of Sox2 in ES undergoing directed differentiation. It will be worthy to profile the remainder of the region to better comprehend the part played by DNA methylation in regulating the activity of this enhancer and Sox2 in differentiated and undifferentiated mouse ES.

## Materials and Methods

### Cell Culture and Neural Differentiation

E14Tg2a (created from mouse strain 129/Ola, kindly provided by Dr. Cristina Tufarelli, University of Nottingham, Nottingham, UK) cells were retrieved from liquid nitrogen and maintained in DMEM (Invitrogen Gibco) containing 10^3^U/ml leukemia inhibitory factor (LIF; Millipore Chemicon), 18% fetal calf serum (SLI), 1 mM non-essential amino acids (Invitrogen Gibco), 100 μM β-mercaptoethanol (βME) (Sigma), 1 mM sodium pyruvate (C_3_H_3_NaO_3_) (Invitrogen Giboc), and 50 U/ml penicillin/streptomycin (Invitrogen Gibco) on gelatinized culture flasks in 5% CO_2_ at 37°C. E14Tg2a were grown at least for three passages if retrieved from liquid nitrogen before setting up the differentiation. Cells were grown to 70–80% confluence before passaging with medium change every day. For passaging, trypsin-ethylenediaminetetraacetic acid (0.25% TE; Invitrogen Gibco) was added to each flask. Then re-suspended the disaggregated cells in fresh medium after counting (1 × 10^6^ cells per ml in to a new gelatinized T-25) and left to grow. This process was repeated and continued until differentiation was set up.

E14Tg2a cells were differentiated into neural lineage as described earlier (Bibel et al., 2004, 2007). Briefly cells were first grown in EB medium in non-adherent petri dishes (Greiner, Cat. No. 633102) to form embryoid bodies (EB) in suspension for 4 days. This was continued with the addition of 0.5 μM all-*trans*-retinoic acid (Sigma) in the EB medium after day four to induce neural precursor formation. At day eight, EBs were collected after low-speed centrifugation, disaggregated with trypsin as described before and counted. 4 × 10^6^ cells were plated in 3ml of DMEM/F12 (Invitrogen Gibco) supplemented with N2 (Invitrogen Gibco), 1 mM Glutamax (Invitrogen Gibco) and penicillin/streptomycin (50 U/ml; Invitrogen Gibco) in each well. The process was repeated after 2, 24, and 48 h post-plating. Then substituted with neurobasal medium (Invitrogen Gibco) with B27 (Invitrogen Gibco) and 50 U/ml penicillin/streptomycin. The culturing of cells were continued for 28 days.

### RNA Extraction and Reverse-Transcription PCR

For RNA extraction, cells at different pre-selected time-points were collected after trypsinization, and resuspended in TRI-reagent (Sigma) after washing with 1X PBS. RNA was extracted as per manufacturer’s protocol and resuspended in RNA grade water and quantified by NanoDrop (Thermo scientific). To remove any residual genomic DNA, DNase treatment was applied after RNA extraction using 200U of DNase enzyme (Roche) as per their protocol. To analyze gene expression, the extracted RNA was then reverse transcribed to cDNA using Expand RT (Roche) according to their protocol with random primers. Polymerase chain reaction was performed by gene-specific primers and cDNA as template. PCR reaction was consisted of 1X Thermopol buffer (NEB), 25 μM forward primer, 25 μM reverse primers, 250 mM dNTPs (Promega) and Thermopol Taq Polymerase (0.5 U/μl; NEB). PCR machine was programmed for: 3 min at 95°C, 40 cycles of 95°C for 30 s, 60–64°C annealing temperature depending on the primer for 30 s, 30 s at 72°C; and a final extension step of 72°C for 5 min.

### Genomic DNA Extraction and Methylation-Sensitive PCR (MS-PCR)

PBS (1X) was used to wash the harvested cells from selected time-points after trypsinization. These washed cells were then re-suspended in lysis buffer (2 ml) and left at 37 degree centigrade for overnight incubation. Cell-lysis method (Sambrook et al., 1989) was used for DNA extraction making use of phase lock tubes (Qiagen). Forty units of *Msp*I and *Hpa*II (BioLabs) restriction enzymes were applied to digest the extracted genomic DNA (1 μg) as per manufacturer’s protocol. Using phenol-chloroform, the samples were re-extracted following digestion. DNA pellets thus obtained were mixed with suitable volume of deionised water and quantified by Nanodrop. PCR using digested genomic DNA as template was carried out as detailed earlier choosing primer-specific annealing temperatures.

### Bisulfite Sequencing

Bisulfite conversion of genomic DNA was done with EZ DNA Methylation-Gold kit (Zymo research) following their protocol for methylation analysis. The converted DNA was then used as template for PCR amplification employing primers specific for SRR2. The band obtained was excised from post-PCR agarose gel using gel extraction kit (Qiagen) as per their instructions. Nanodrop was used to quantify the extracted product and subsequently Sanger-sequenced. Chromas software (Technelysium Pty Ltd.) was used to examine the DNA sequence chromatograms and BiQ analyzer (Bock et al., 2005) for DNA methylation analysis.

### Immunocytochemistry

The wells of culture plate were covered with glass cover slips prior to seeding cells in the differentiation medium after dissociation of EBs. 4% (w/v) paraformaldehyde was added to fix the cells growing on cover slips at room temperature to perform immunocytochemistry. These were subsequently stored in PBS (pH 7.4) at 4 degree centigrade until further analysis. Anti-Sox2 (abcam ab97959) and anti-Sox9 antibodies (Millipore AB5535) were added to the cells for staining. Briefly, fixing of cells was followed by washing with PBS buffer plus 0.1% Tween-20 (v/v) for 10 min with shaking. Afterward, blocking was done at room temperature for 1 h using block solution. Cells were then left to incubate overnight at 4°C with primary antibody, i.e., Anti-Sox2 and Anti-Sox9 diluted in block solution. Cover slips containing cells were washed next day with PBS plus Tween-20 thrice for 15 min each with shaking. The washed cells were incubated for 1 h diluted in same block as that of primary antibody with FITC-conjugated secondary antibody (Vector Laboratories DI-1488-1.5) in dark at room temperature. Special care was taken after completion of this step to minimize light exposure. Cover slips were washed again after incubation with secondary antibodies with PBS-Tween-20 thrice for 15 min each at room temperature. At the end, cells were placed on glass slide with the help of Vectashield (Vector Laboratories) and kept at 4°C in dark until observation. Nikon Eclipse 90i fluorescent microscope was used to visualize the cells and Volocity 3D image analysis software (PerkinElmer) was used to capture the images.

## Results

### Cells Culture and Differentiation

**FIGURE 1:**
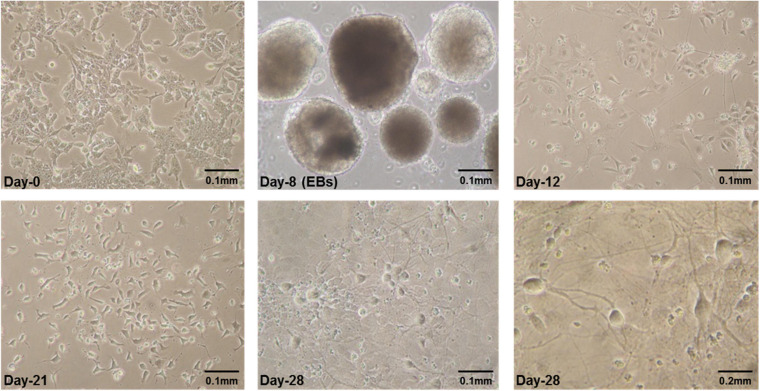
Some representative pictures of mES cells undergoing neural differentiation. Mouse embryonic stem cells (mES) undergoing differentiation. E14Tg2a cells were photographed at different time-points during *in vitro* neural differentiation at 0.1 mm (10X) and 0.2 mm (20X) magnification. Day-0 refers to undifferentiated mES cells before starting differentiation and Day-8 are embryoid bodies (EBs) grown in suspension culture in non-adherent petri plates. Cells having specific neuronal morphology can be seen at day-12 and by day-21 and day-28 majority of cells in culture were differentiated. These are the results from three independent differentiation experiments.

E14Tg2a cells were grown for 8 days in embryoid bodies (EB) medium adding all-trans-retinoic acid (RA) in last 4 days as reported previously (Bibel et al., 2004, 2007). The specific neural morphology was apparent a day after plating of cells obtained from dissociated EBs. After plating, neurites were observed forming dense and tangled networks by day-4 which became thick by second, third and fourth week in culture (Figure 1).

### Characterization of Differentiated Cells by Gene Expression and Immunocytochemistry

**FIGURE 2:**
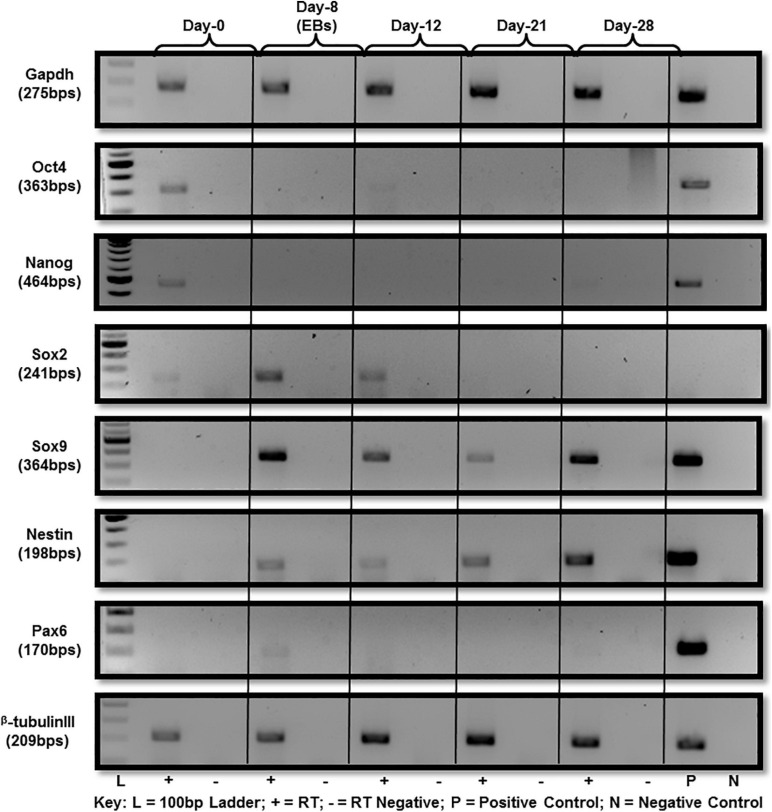
Gene expression analysis of mES during neural differentiation time-course. Gene expression analysis of mES subjected to neural differentiation. RNA extracted from E14Tg2a cells grown in neural differentiation medium at day-0, day-12, day-21, and day-28 was reverse transcribed using random primers. Pluripotency markers, i.e., Oct4, Nanog and Sox2 were found to be down-regulated after day-8 (Supplementary Figure 4). Neural-markers, i.e., Sox9, Nestin, Pax6, and beta-tubulin III were up-regulated in the cells undergoing differentiation after day-8. Gapdh was used as endogenous control. Each PCR was performed in triplicates from three independent differentiation experiments.

A series of established markers were selected for further detailed characterization of the differentiated cells beside cellular morphology. These markers were chosen in accordance with previous studies reporting *in vitro* differentiation of ES into neural lineage for molecular expression analysis at RNA and protein level. Those selected for analysis were Sox2, Sox9, Nestin, Pax6, and beta-tubulin III. ES pluripotency markers, i.e., Oct4, Nanog and Sox2 were all expressed at day-0 (Figure 2). Down-regulation after day-0 was noted for both Oct4 and Nanog with a little expression of Oct4 persisting until day-12 as cells differentiate (Figure 2). Sox2 expression was observed by day-12 of differentiation and no expression afterward (Figure 2) at RNA level.

**FIGURE 3:**
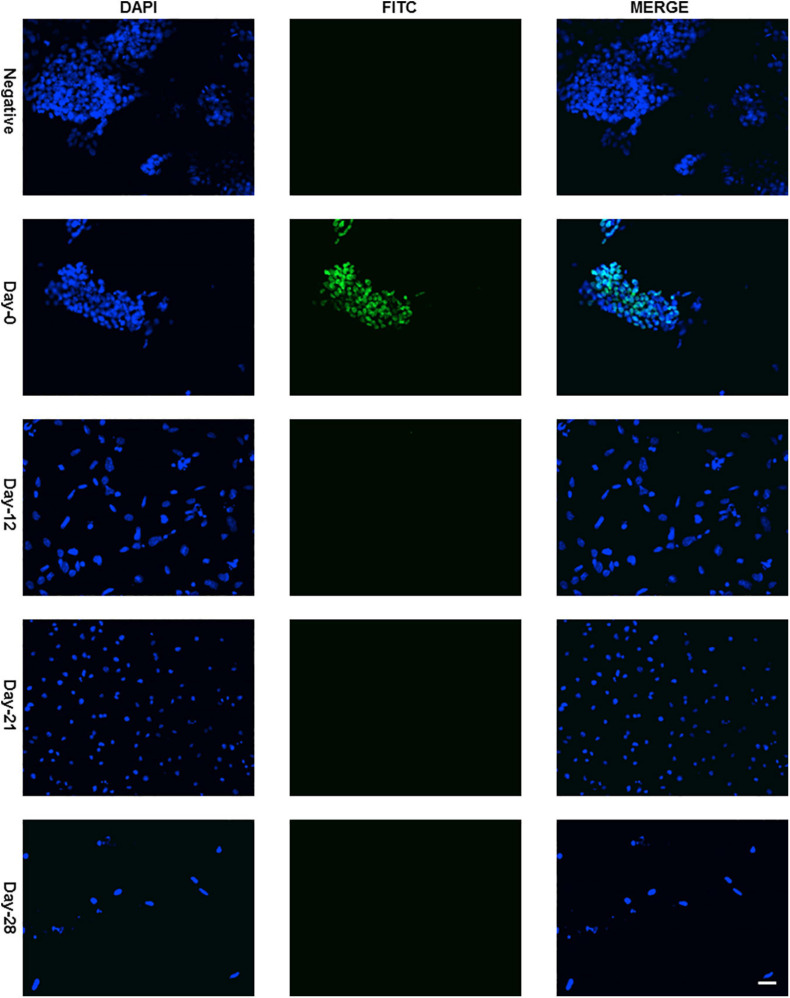
Immunocytochemistry with Anti-Sox2 Antibody. Almost all cells of undifferentiated ES cell colony were found positive with nuclear localization (Figure 4 with higher resolution at day-0) and no expression was detected at day-12, day-21, and day-28. Dapi (4′,6-diamidino-2-phenylindole) was used to stain the nucleus and anti-rabbit IgG secondary antibody (FITC-conjugated) against primary anti-Sox2 antibody was used (scale bar is 32 μm) for visualization.

**FIGURE 4:**
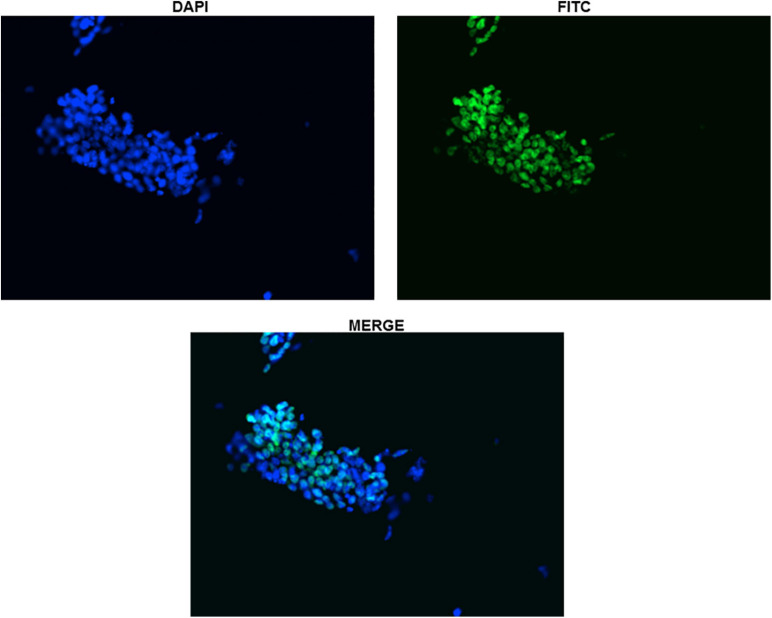
Immunocytochemistry with Anti-Sox2 Antibody (Day-0). The nuclear localization of Sox2 antibody has been shown for clarity in undifferentiated ES cells. All the cells can be seen clearly expressing Sox2.

Protein expression of Sox2 was also investigated by immunocytochemistry and all the undifferentiated cells in ES colony were found positive as shown in Figures 3, 4. There was no Sox2 protein expression at day-12 and beyond (Figure 3). The RNA expression of Sox2 at day-12 could be attributed to the presence of transcribed product but no translated functional protein product.

RNA expression analysis of Sox9 revealed notable up-regulation at day-8 EB stage (Figure 2) followed by down-regulation. By day-28 after plating of cells in neural differentiation medium, it was up-regulated again. However, no Sox9 protein expression was observed along the entire differentiation time-course starting from day-0 to day-28 (Supplementary Figures 1, 2).

Nestin protein is a known marker for neural stem cells (NSCs) characterization. Up-regulation of nestin was observed at day-8 (EBs) and that expression was seen until day-28 of differentiation (Figure 2). Since nestin is considered a marker of NSCs, hence this late expression may be accounted for by a subpopulation of progenitor cells.

Pax6 is also a marker of neural progenitors and was observed to be up-regulated in EBs after 8 days of differentiation which should presumably be rich in neural precursors after RA treatment. Pax6 expression was not observed beyond day-8 (Figure 2). A member of the tubulin class of proteins, beta-tubulin III has long been used as a classic marker to establish *in vitro* neural differentiation. Beta-tubulin III expression was up-regulated post day-8 of differentiation which remained until day-28 (Figure 2).

In summary, all of the morphological and molecular findings described above proved that ES were differentiating and, results obtained were comparable to the previous studies. Gene expression analysis of neural markers showed their upregulation, i.e., Sox9, Nestin and Pax6 in day-8 EBs in contrast to no expression seen in undifferentiated cells at day-0 undifferentiated cells. And there was visible upregulation of beta-tubulin III, a marker extensively used to characterize differentiated neural cells.

### Methylation Sensitive PCR (MS-PCR) of Sox2-SRR2

**FIGURE 5:**
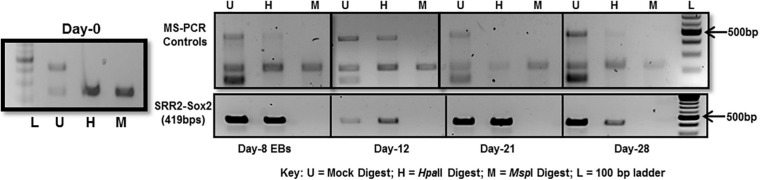
DNA methylation analysis of Sox2-SRR2 by MS-PCR. Top panel shows multiplex PCR for control primers at all time-points, i.e., day-0, day-8, day-12, day-21, and day-28 indicating complete digestion of the genomic DNA. Lower panel is Sox2-SRR2 amplified product in H lane indicative of DNA methylation at all the sites in the fragment as no product was observed in M lane. Any product in the M lane would have indicated the presence of undigested genomic DNA thus giving false positive results. This analysis was performed in triplicates from three independent differentiation experiments.

The whole SRR2 region (about 400 bps) of Sox2 was observed methylated at chosen time-points, i.e., day-8, 12, 21, and 28 in differentiated cells 4 and unmethylated in undifferentiated cells at day-0 as shown in Figure 5. This PCR is dependent on the differential digestion of genomic DNA by *Msp*I and *Hpa*II restriction enzymes which are isoschizomers (same target site CCGG). *Msp*I is able to cleave its target sequence irrespective of DNA methylation while *Hpa*II would not cleave its methylated recognition sequence. Three PCR reactions were therefore run in parallel for these three set of samples: Uncut (U) refers to mock digested genomic DNA which did not receive any enzymatic treatment, M refers to genomic DNA digested with *Msp*I and H is for genomic DNA digested with *Hpa*II restriction enzyme as shown in Figure 5.

Multiple controls were included in the PCR reaction to check the quality of DNA and, to ensure the efficiency, specificity and success of the restriction reactions. One set of control primers was targeted at the regions of digested DNA without any *Msp*I/*Hpa*II sites, so there should always be amplification if the template DNA was of good quality. Additional two sets of primers targeted at those sites that are established to be always unmethylated and methylated in undifferentiated and differentiated cells. For constitutively unmethylated sequence, a region of DNA was selectively amplified using mAprt primer which has been reported to contain *Msp*I/*Hpa*II sites but remain methylation free during differentiation in mouse cells (Macleod et al., 1994). Insulin like growth factor receptor differentially methylated region 2 (Igf2R-DMR2) from mice was selected as constitutively methylated control. This region becomes methylated during oogenesis and then remains methylated (Feil et al., 1994).

As can be seen in Figure 5-top panel that region of mAprt known to be unmethylated did not amplify in both *Msp*I and *Hpa*II digested DNA samples at any time-point in differentiated cell population. This indicated that enzymatic digestion was not partial and any product thus obtained with primers specific for Sox2 regulatory region 2 (SRR2) would be due to their methylation. SRR2 was observed methylated at day-8, 12, 21, and 28 as only H (*Hpa*II) lane showed amplified product and no amplification was seen in M (*Msp*I) lane following PCR. There was no amplification in undifferentiated cells at day-0 (Figure 5). This means that all of the CpG sites in the whole of 419 bps long SRR2 have become methylated during differentiation. But since this technique relies on the amplification of digested fragments, it is entirely possible that sticky ends generated after digestion might join together during the amplification process and product is obtained even when some of the CpG sites are not methylated. MS-PCR therefore is primarily used to screen the presence and absence of DNA methylation in the sequence of interest and do not provide any information about which of the individual CpGs have become methylated and to what extent. Hence, DNA sequencing after bisulfite conversion was performed to actually assess the sites and level of methylation.

### Direct DNA Sequencing After Bisulfite Conversion

**FIGURE 6:**
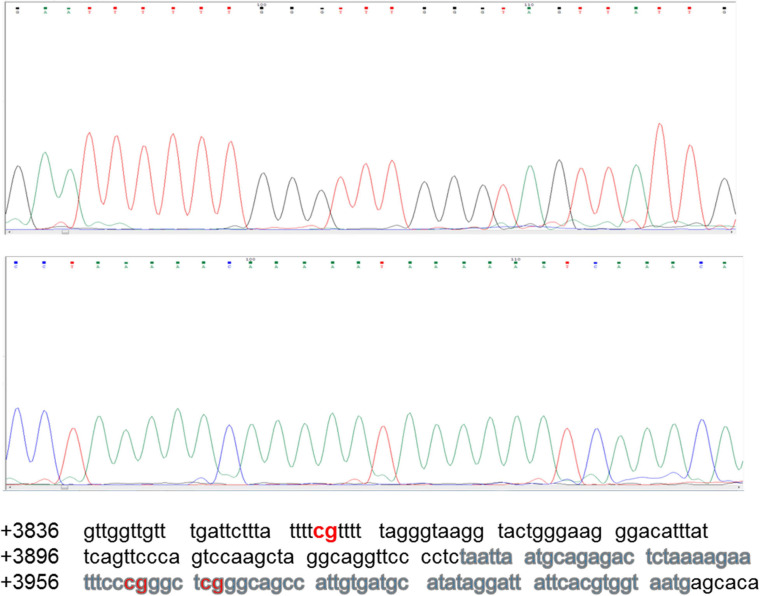
Sequencing chromatogram of Sox2-SRR2. SRR2 spans from +3641 to +4023 downstream of the gene relative to transcription start site (GeneBank ID: NG_051227.1). Part of the sequence which was analyzed by direct DNA sequencing after bisulfite conversion and contained 81bp core enhancer sequence (+3931 to +4011) highlighted in gray is shown here at the bottom. Sequencing chromatogram shows the three unmethylayted CpGs of which two are in core enhancer region, i.e., +3961 and +3967.

The region analyzed by direct DNA sequencing contained three CpG dinucleotides of which two are part of core enhancer region and no methylation was observed (Figure 6). The CpGs analyzed are located downstream, i.e., +3860, +3961, and +3967 from transcription start site (GeneBank ID: NG_051227.1).

**FIGURE 7:**
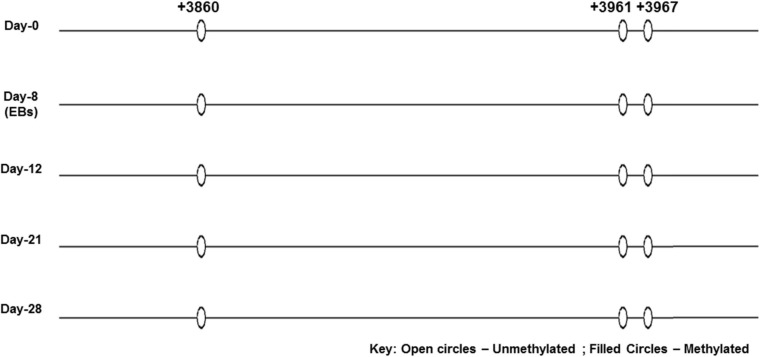
Lollipop diagram of CpGs analyzed by direct DNA sequencing. Lollipop diagram generated from BiQ Analyzer after bisulfite sequencing. The three CpGs in the enhancer region of SRR2 appear to be unmethylated in undifferentiated ES cells and after differentiation at day-8, day-12, day-21, and day-28. This was done in duplicates from two independent differentiation experiments.

None of the CpG analyzed at any of the time-point found methylated. A clear T can be seen in sequencing chromatogram shown in Figure 5 at positions +3961 and +3967 and a C in opposite strand at position +3860. Apparently this seems contradictory that MS-PCR has shown the presence of DNA methylation and direct DNA-sequencing after bisulfite conversion failed to found methylation. As detailed in previous section that re-joining of the digested fragments due to sticky ends post-digestion could still give amplified product and therefore further analysis is needed to rule out the presence and absence of methylation at each CpG site. It could be possible that these sites become methylated between the analyzed time-points and/or remain methylation free while the other sites become methylated. Figure 7 presents the lollipop view generated from DNA methylation analysis software BiQ analyser (Bock et al., 2005) and clearly shows no methylation at any of the CpG site investigated.

## Discussion

For Epigenetic mechanisms such as DNA methylation of CpG dinucleotides, alteration of histone tails mostly by acetylation, methylation and phosphorylation, regulatory RNAs and polycomb group proteins (PcG) together with transcription factors control and influence cellular commitment and differentiation (Jaenisch and Young, 2008). The interplay among these factors leads to generation of cell-type and tissue-specific transcriptomes and epigenomes governing their phenotype as demanded by cellular environment despite shared genotype. The resultant interactions ensure reversible repressive expression of genes of earlier development at later stage of development; and/or irreversible silencing of pluripotency associated genes after cellular commitment and differentiation (Morgan et al., 2005; Reik, 2007). ES cells derived differentiated cell types could provide a better and suitable mean for *in vitro* studies and profile cell-specific epigenomes including DNA methylation as cells differentiate. The knowledge obtained through such studies could provide an insight into the regulatory framework operating in the cells influencing cellular commitment which will ultimately help to understand the neural development and, a possible cure for neurodegenerative disorders affecting millions worldwide.

Given the central place of Sox2 transcription factor in pluripotency hierarchy, a good number of studies have pointed out the regulatory function of Sox2 in embryonic stem cells (Boyer et al., 2005; Smith et al., 2016). But still much has to be learnt about the epigenetic regulation of the Sox2 itself. The work presented here has mainly focused on SRR2 of Sox2 in mouse ES. It has been shown previously that this region exerts its enhancer activity in ES cells and NS cells but do not function when cells differentiate (Miyagi et al., 2004). Embryonic stem cells were subjected to transition from an undifferentiated state to differentiated state and DNA methylation of SRR2 was investigated at selected time-points during this process. This time-course analysis was performed to identify the potential correlation of Sox2 expression with DNA methylation if/when the onset of methylation takes place during differentiation.

Mouse ES cells (E14Tg2a) were first grown in non-adherent bacterial dishes for a total of 8 days to form embryoid bodies (EBs) where retinoic acid (RA) was added in culture media in the last 4 days as reported previously (Bibel et al., 2004, 2007). The existence of cells exhibiting specific neural morphology was clearly evident under microscope a day after plating of cells obtained from dissociation of EBs. Thick and intertwined networks of neurites with typical neuronal morphology were observed as the differentiation progressed and, can be clearly seen in Figure 1. Although the majority of the differentiated cells looked like neurons, there were still other cell types present. Neural differentiation of mouse embryonic stem cells has been reported most often to result in generation of various types of neuronal and non-neuronal cell types in the cultures (Stavridis and Smith, 2003). Some molecular markers of neural lineage were used to characterize the differentiated cells such as Sox2, Nestin, Sox9, Pax6, and beta-tubulin III. Neuroectodermal specification and commitment has been reported to be coordinated and regulated by Sox2 together with other proteins of SoxB family (Pevny and Placzek, 2005). More specifically, Sox2 is required to keep up the identity of neural progenitors (Graham et al., 2003) and, once these cells have become committed to differentiate, expression of Sox2 is down-regulated. RNA expression analysis of Sox2 in these experiments showed down-regulation at day-8 EBs which continued to decline until day-12 and no expression was seen beyond that.

Sox2 protein expression too was investigated by immunocytochemistry in addition to gene expression and, only undifferentiated cells in ES colony were observed to be positive (Figure 3). There was no Sox2 protein expression at day-12 contrary to RNA expression, which could possibly be due to the presence of yet to be transcribed RNA transcripts but post-transcriptional gene-regulation not leading to the formation of protein. Post-transcriptional gene silencing mechanisms involving short microRNAs, small interfering RNAs and long non-coding RNAs are now well-known regulatory switches specifically in embryonic stem cell differentiation (Dinger et al., 2008; Tay et al., 2008). Moreover, Sox2 gene has also been shown to be incorporated inside an intron of a long non-coding RNA. This RNA is transcribed in the same orientation as that of Sox2 gene and has been proven to have regulatory roles during vertebrate development (Amaral et al., 2009).

Gene expression analysis revealed notable up-regulation of Sox9 at day-8 EB stage of differentiating cells (Figure 2). Sox9 is documented to be up-regulated before gliogenesis resulting in shift of neural progenitor’s potential from neurogenic to gliogenic and, disappears from oligodendrocyte lineage in terminally differentiated cells (Stolt et al., 2003). Retinoic acid treated ES cells have been reported to form precursor cells having radial glial cells characteristics that eventually differentiate into glutamatergic neurons (Bibel et al., 2007). After day-8 EB stage, Sox9 was observed to be down-regulated but again up-regulated in cells growing in neural differentiation medium at day-28. However, there was no expression of Sox9 protein at any time-point chosen for analysis from differentiation time-course (Supplementary Figures 1, 2, where Figure 1 is positive control for Sox9 discounting the possibility of non-functional assay). It is possible that some post-transcriptional gene-regulatory mechanisms such as microRNAs are involved so mRNA is present but no protein. It has been reported that microRNA-124 (miR-124) is involved in suppression of Sox9 protein synthesis but mRNA expression remains unaltered in adult neurogenesis (Cheng et al., 2009).

Nestin and Pax6 as markers of neural progenitors were found up-regulated in EBs (day-8). Pax6 has been reported to have dynamic expression changes in ES cells undergoing neuronal differentiation with low expression in undifferentiated embryonic stem cells and high level of expression in NSCs and again low to no expression in differentiated neuronal cell types (Gao et al., 2011). RNA expression analysis in our experiments too showed that Pax6 was not expressed in undifferentiated ES cells at day-0 but very slightly up-regulated in EBs at day-8 of differentiation which should be enriched in neural progenitors after retinoic acid treatment in culture medium (Figure 2). Then no Pax6 expression was seen at RNA level after day-8 of differentiation (Figure 2) consistent with earlier studies reporting no detectable Pax6 RNA after day-12 in ES cells differentiated to neuronal cell types under similar culture conditions (Bibel et al., 2004, 2007). Beta-tubulin III as classical neuronal marker in was markedly up-regulated in differentiated cells (Figure 2). After confirmation of the differentiation of the ES cells, DNA methylation analysis was carried out.

The whole of the SRR2 region when examined by MS-PCR appeared to be methylated at all selected time-points except in undifferentiated (day-0) embryonic stem cells where Sox2 expression is high (Supplementary Figure 3). As detailed earlier that the MS-PCR employed was limited to methylation analysis only at *Msp* sites, bisulfite sequencing was then carried out to analyze individual CpGs in core enhancer region. Bisulfite sequencing is still considered as gold-standard for sequence-specific DNA methylation analysis and widely employed (Harrasi et al., 2017). The core enhancer sequence of SRR2 sequence is 81bp long and contains a binding site for Sox2/Oct4 complex (Tomioka et al., 2002). This study thus analyzed three CpGs of the SRR2 enhancer, of which two are part of the 81 bp core enhancer region having a binding site for Oct4/Sox2. They were all found to be unmethylated at selected time-points during differentiation hinting toward the possibility of transient methylation at these sites or may remain free of methylation under conditions used for differentiation. SOX2-SRR2 together with SOX2-SRR1 has been investigated for epigenetic regulation in human neural progenitors. These two enhancers, i.e., SRR1 and SRR2 show differential DNA methylation and histone H3 acetylation affecting expression level of SOX2 in different cell types during human neural progenitor differentiation into astrocytes and neurons (Sikorska et al., 2008). Methylation of human SRR2 enhancer particularly at a highly conserved site +4250, correlated with silencing of SOX2 expression in post-mitotic neurons. This showed that differential DNA methylation of these two enhancers of SOX2 regulate gene expression transiently and permanently in neural precursors and terminally differentiated cells and modulate neurodevelopment.

But SRR2 has not been yet profiled for DNA methylation in embryonic stem cells (ES) and during their growth in neural differentiation medium. The present study looked at three individual CpGs present in core enhancer region of SRR2 in differentiating neural cells obtained from embryonic stem cells. These CpGs were found to be free of methylation in both undifferentiated ES cells and differentiated cells suggesting possible involvement of other gene silencing mechanisms independent of DNA methylation. The asynchronous division of cells in culture could also contribute to masking low level of methylation as has been reported previously that only post-mitotic neurons use the DNA methylation of SRR2 to silence the SOX2 expression (Sikorska et al., 2008). It is also possible that CpGs other than those analyzed here could have become methylated during differentiation since MS-PCR analysis is limited to *Msp* sites as detailed earlier. Hence future work extending the methylation analysis to all CpG sites present in SRR2 region is needed. Additionally, chromatin structure around the region being examined for methylation should also be investigated to better understand the role of this region in regulation of Sox2 expression when cells become committed and differentiate.

## Conclusion

This study profiled three CpG sites of SRR2 regulatory region of Sox2 in embryonic stem cells undergoing *in vitro* neural differentiation and found them unmethylated. Given the central importance of epigenetic regulation during differentiation and cellular commitment, analysis of the regulatory regions of key modulator genes such as Sox2 will provide a required mechanistic insight into gene regulation and specifically how they contribute toward cell fate decisions and switch between different cell types.

## Data Availability Statement

The raw data supporting the conclusions of this article will be made available by the authors, without undue reservation.

## Author Contributions

SB and KK: conceptualization and visualization. SB: methodology, formal analysis, and writing—original draft preparation. SB, MK, and KK: writing—review and editing. KK: supervision. SB and MV: project administration and funding acquisition. All authors have read and agreed to the published version of the manuscript.

## Conflict of Interest

The authors declare that the research was conducted in the absence of any commercial or financial relationships that could be construed as a potential conflict of interest.
