# Supplementary material for: Neural Differentiation of Mouse Embryonic Stem Cells—An in vitro Approach to Profile DNA Methylation of Reprogramming Factor Sox2-SRR2
Source: Front Genet. 2021 Mar 22;12:641095. doi: 10.3389/fgene.2021.641095 (PMC8019947; doi:10.3389/fgene.2021.641095)
Supplement: Supplementary file 1 [file Data_Sheet_1.PDF]

# *Supplementary Material*

## 1 Supplementary Figures

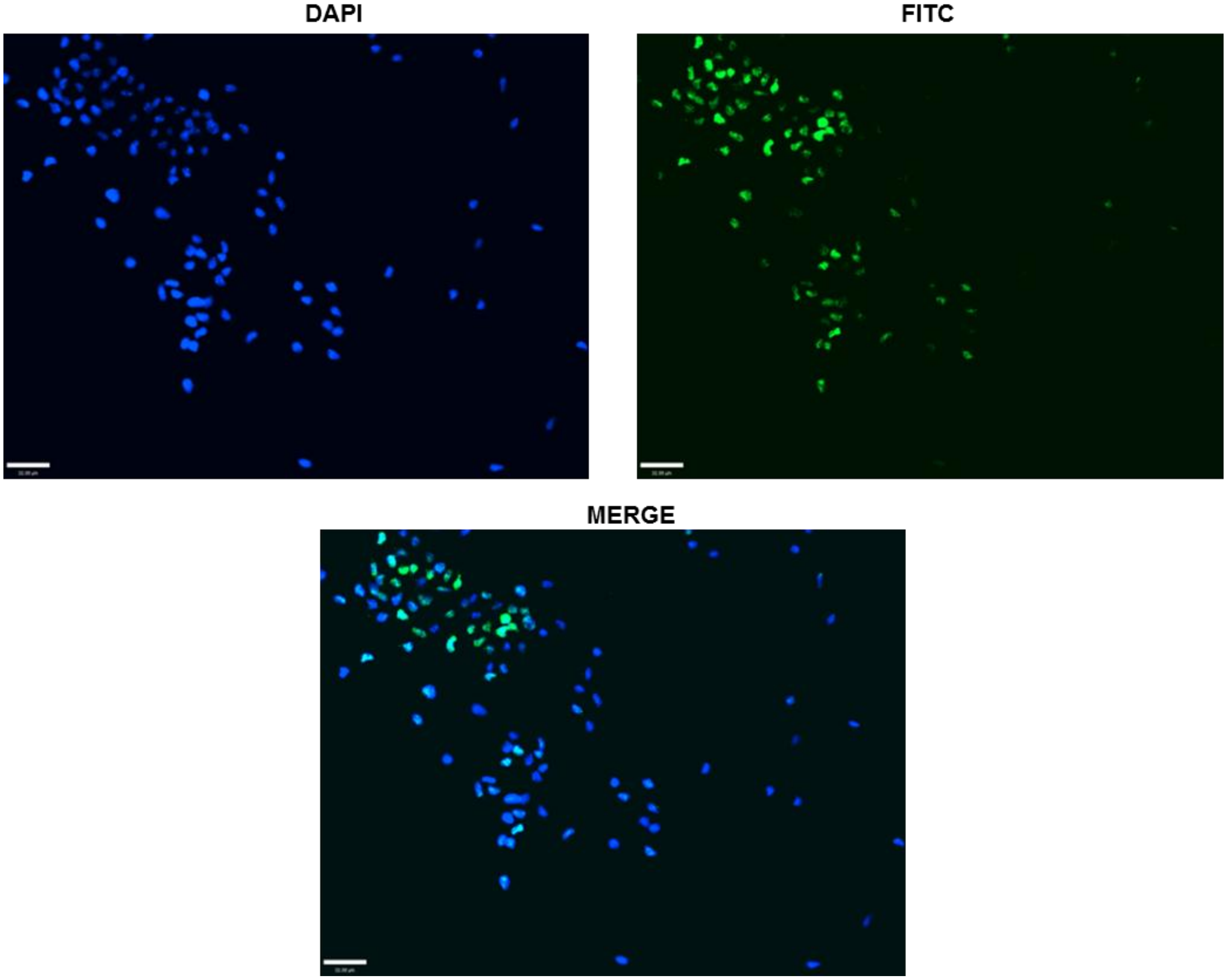


**Supplementary Figure 1**: Primary mouse neural stem cells (NSCs) isolated from lateral ventricle of brain (kindly given by a fellow researcher Dr. Stephanie Strouhbecker, Centre for Biomolecular Sciences, University of Nottingham) are shown here stained with anti-Sox9 antibody demonstrating the nuclear presence without non-specific background. Sox9 has been reported to be essential for NSC formation and maintenance and, is routinely used in the laboratories as marker for NSCs (Scott *et al*., 2010). Accordingly, these cells are chosen as positive control to validate the anti-Sox9 antibody. Nucleus was stained with Dapi and anti-rabbit IgG secondary antibody (FITC conjugated) against anti-Sox9 primary antibody was used for visualization (scale bar is 32µm).

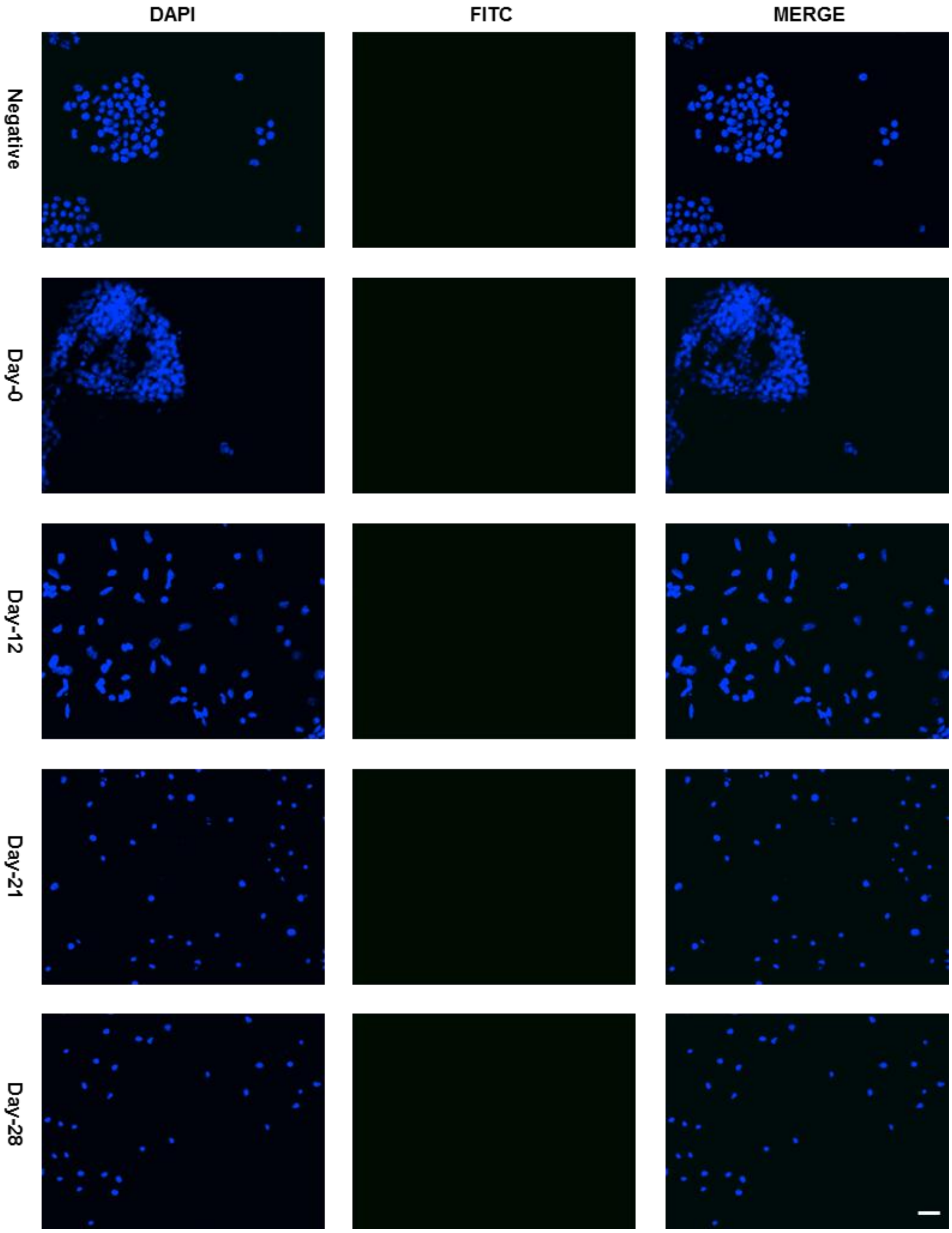


**Supplementary Figure 2:** Immunocytochemistry with anti-Sox9 antibody. Some representative images of E14Tg2a cells undergoing neural differentiation at day-0, day-12, day-21 and day-28 after performing immunocytochemsistry (scale bar is 32µm). Nucleus was stained with Dapi and anti-rabbit IgG secondary antibody (FITC conjugated) against anti-Sox9 primary antibody was used for staining.

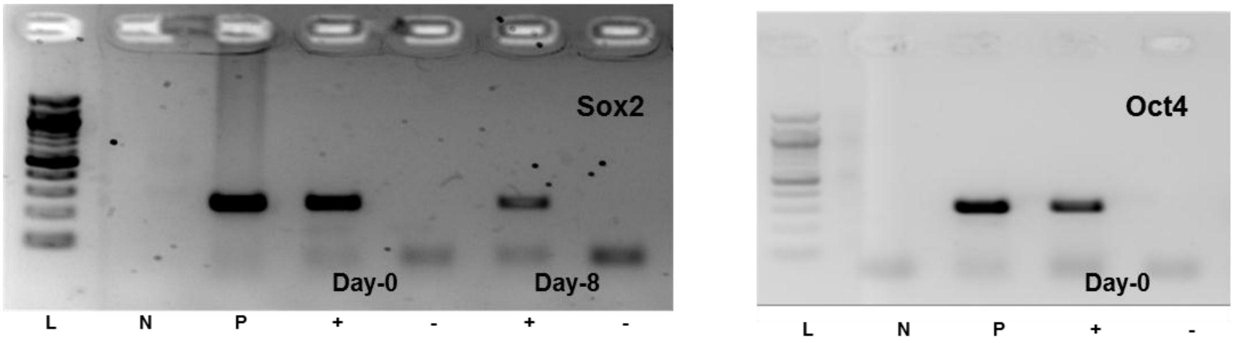


**Supplementary Figure 3**: Expression of Sox2 and Oct4 in undifferentiated embryonic stem cells (Day-0) together with their positive controls. They were found to be expressed in all of the assays carried out for gene expression analysis at different stages along the differentiation time course. These are shown here to supplement the data shown in Figure 2 in main text where expression of both Sox2 and Oct4 was observed to be low perhaps due to the variations in the amount of RNA input. 'L' in each figure represents 100bp DNA Ladder; '+' means RT and '-' means RT Negative for respective time-point; 'P' is positive control and 'N' is PCR negative control.

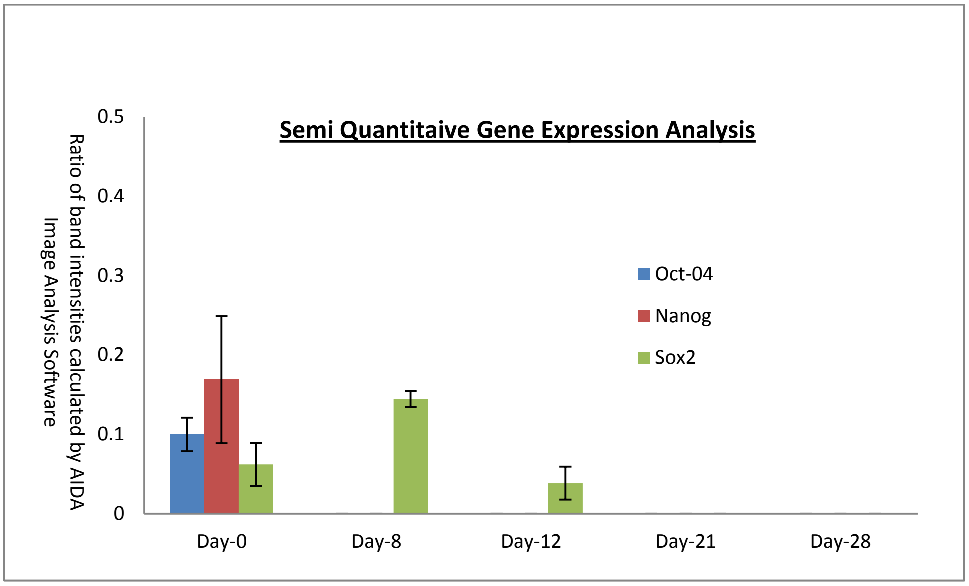


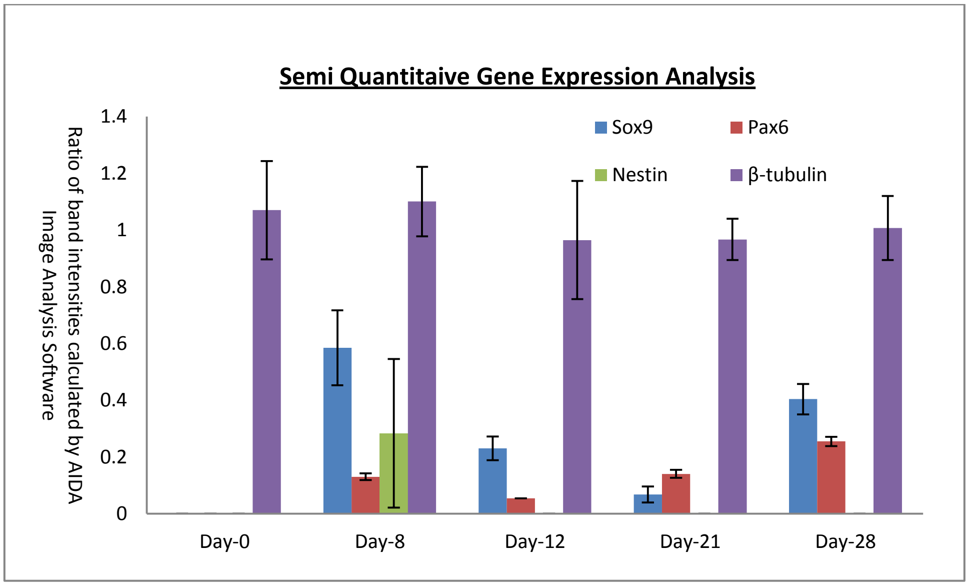


**Supplementary Figure 4**: Expression of pluripotency markers (Oct4, Nanog and Sox2) and neural markers (Sox9, Pax6, Nestin, β-tubulin III) was semi-quantified by taking the gel-band intensities using the AIDA image analysis software (Elysia-Raytest, Germany). The data is presented here as mean ±SEM.
